# Magnetic Resonance Imaging in Subacute Sclerosing Panencephalitis: Two Case Reports and Review of Literature

**DOI:** 10.7759/cureus.19161

**Published:** 2021-10-31

**Authors:** Saumitra Barthwal, Narinder Kaur, Ravinder Kaur, Ruma Zaidi, Shivani Randev

**Affiliations:** 1 Radiodiagnosis, Government Medical College and Hospital, Chandigarh, IND; 2 Pediatrics, Government Medical College and Hospital, Chandigarh, IND

**Keywords:** sspe, electroencephalogram, magnetic resonance imaging, measles, subacute sclerosing panencephalitis

## Abstract

Subacute sclerosing panencephalitis (SSPE) is a slowly progressive neurodegenerative disease caused by the measles virus. This study investigated the magnetic resonance imaging (MRI) findings in SSPE by retrospective review of two cases diagnosed by typical periodic electroencephalographic (EEG) features, clinical symptoms and elevated measles antibody titre in cerebrospinal fluid (CSF). MRI revealed lesions of high signal intensity on T2-weighted images involving the periventricular or subcortical white matter in both the patients. Both these patients were given sodium valproate for seizures. However, the disease proved fatal in both these cases eventually.

## Introduction

Subacute sclerosing panencephalitis (SSPE) is a rare progressive inflammatory neurodegenerative disease of childhood and adolescence caused by immune resistant measles virus following an asymptomatic period of six to eight years [1,2]. Patients clinically present with personality changes and altered mentation which progresses to stereotypic attacks of myoclonic jerks/atonia. Usually, the disease follows a progressive course proving fatal within one to two years [1].

The annual incidence of this disease varies among the developing nations with an annual incidence in India at around 21 per million population, in comparison to an incidence of 2.4 per million population in the Middle East [3].

There is limited literature available on the imaging findings in rarely encountered disease of SSPE. The authors present a retrospective analysis of magnetic resonance imaging (MRI) findings on 1.5 Tesla MRI scanner (Achieva 1.5 T, Philips, Netherlands) in two children presenting with typical clinical features and electroencephalographic (EEG) changes of SSPE accompanied with an elevated cerebrospinal fluid (CSF) titre of measles antibodies.

## Case presentation

Case report 1

**Figure 1:**
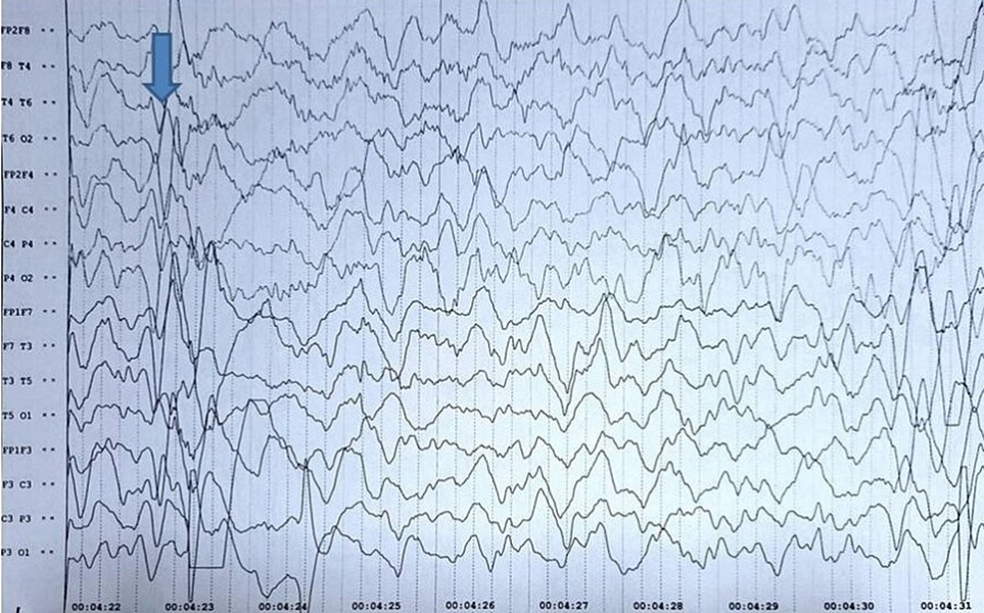
Electroencephalogram (EEG) Characteristic intermittent bursts of generalised discharges (blue arrow) lasting one to three seconds consistent with subacute sclerosing panencephalitis (SSPE).

An 8-year-old male child presented to the paediatrics out-patient department (OPD) with the complaints of sudden jerky body movements and episodes of fall, progressive over six months. There were no similar complaints in the family. The child was not immunised. Physical examination revealed myoclonic jerks, increased tone with normal power in all four limbs and brisk deep tendon reflexes. EEG showed intermittent bursts of generalised discharges (spike, polyspike and wave) lasting for one to three seconds (Figure 1).

CSF sample enzyme-linked immunosorbent assay (ELISA) tested positive for IgG antibodies for measles virus.

**Figure 2:**
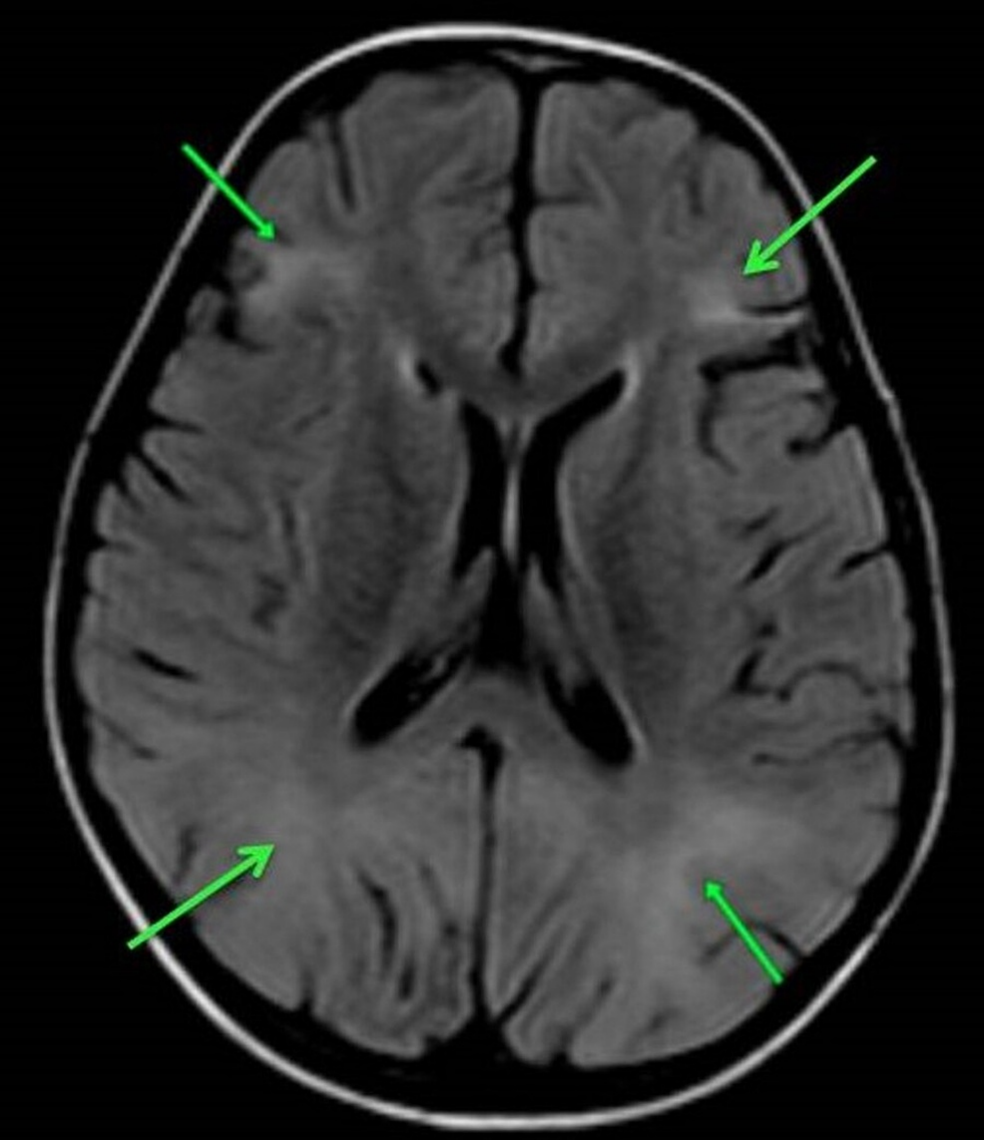
Axial fluid-attenuated inversion recovery (FLAIR) image Confluent hyperintensities involving the subcortical and deep white matter along bilateral frontoparietal regions (green arrows).

Brain MRI examination revealed confluent T2/fluid-attenuated inversion recovery sequence hyperintensities in bilateral (B/L) parietal and frontal white matter (Figure 2).

**Figure 3:**
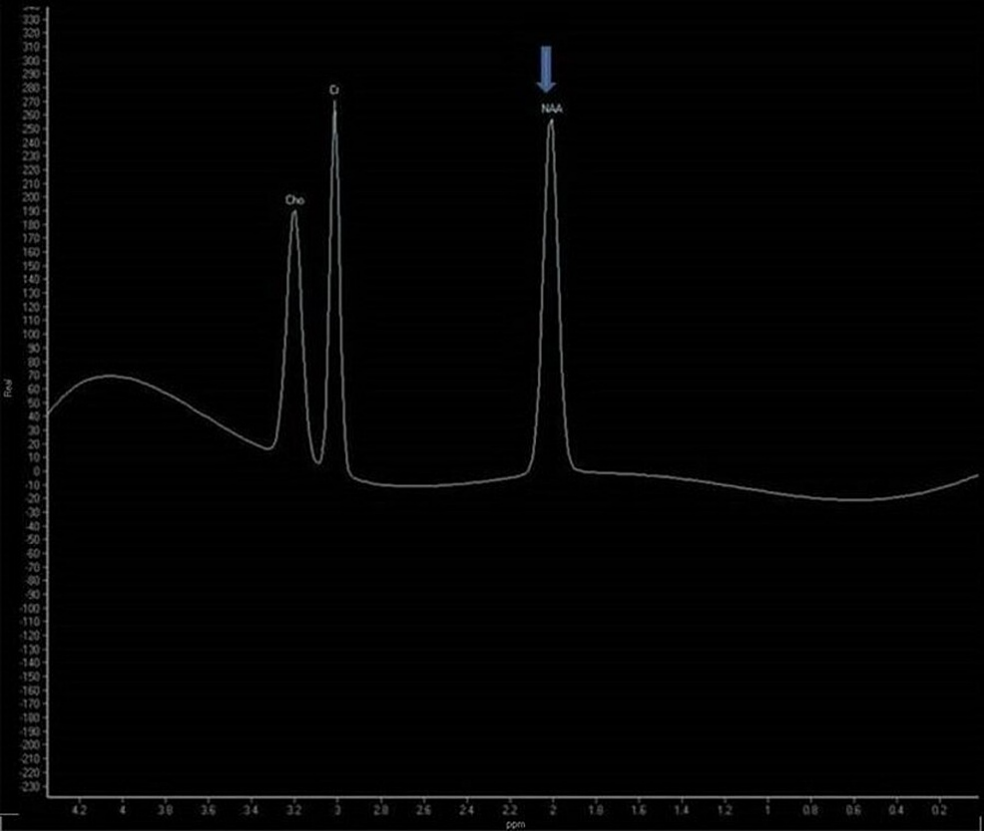
Proton magnetic resonance spectroscopy image (TE: 144 ms) Reduced N-acetylaspartate (NAA) levels (blue arrow) are seen relative to choline (Cho) and creatine (Cr) peaks disrupting the Hunters angle. TE: time to echo.

The lesions showed no diffusion restriction/blooming on diffusion-weighted/gradient images. No post-contrast enhancement was noted. On magnetic resonance spectroscopy (MRS), reduced N-acetylaspartate (NAA) levels were seen (Figure 3).

**Figure 4:**
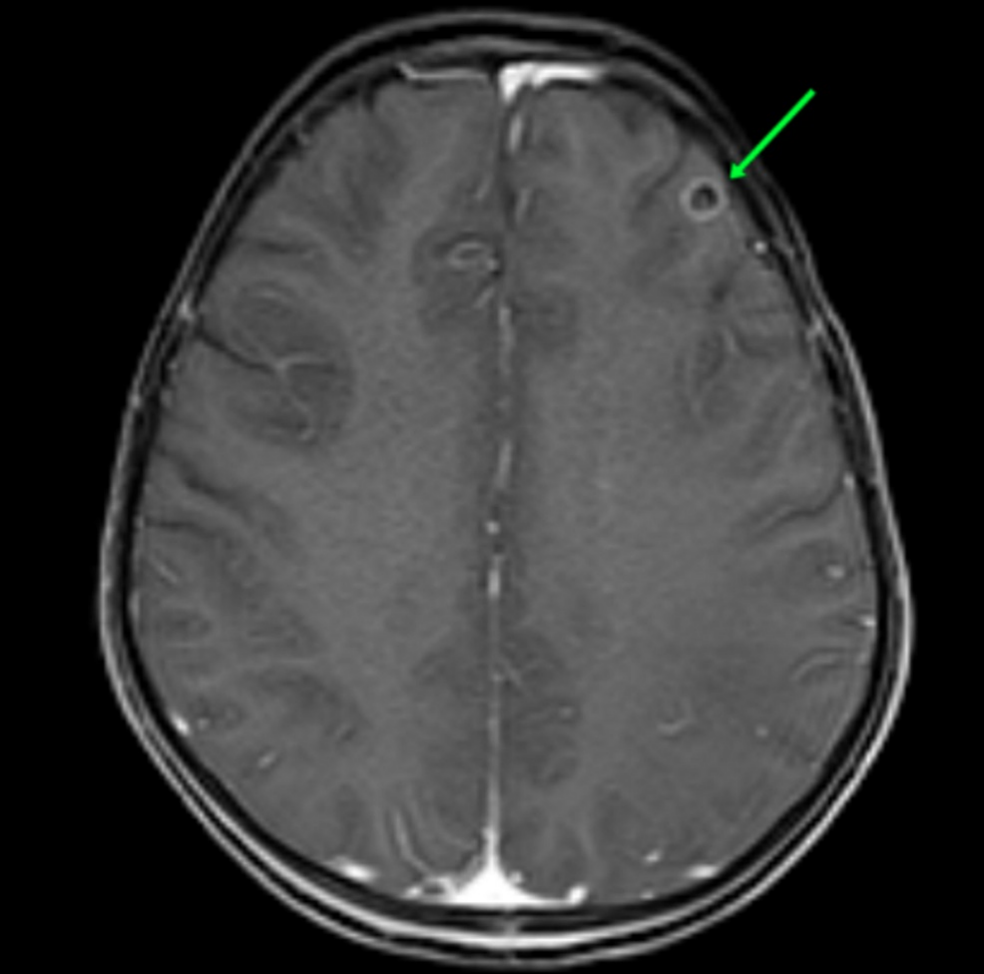
Axial T1-weighted post-contrast image Incidental finding of a small peripherally enhancing neurocysticercosis lesion in left frontal lobe (green arrow).

An incidental neurocysticercosis lesion was also seen in left frontal lobe (Figure 4).

Case report 2

A 15-year-old female child presented to the paediatrics OPD with the complaints of progressive abnormal jerky body movements, inability to walk, episodes of fall and abnormal behaviour over the past 18 months. There was no similar family history. The child was immunised adequately for age. Physical examination revealed myoclonic jerks, increased tone in all four limbs, diminished deep tendon reflexes, power of 5/5 and a normal sensory system evaluation. EEG showed intermittent bursts of generalised discharges (spike, polyspike and wave) lasting for one to three seconds. CSF ELISA test came positive for IgG antibodies to measles virus. 

**Figure 5:**
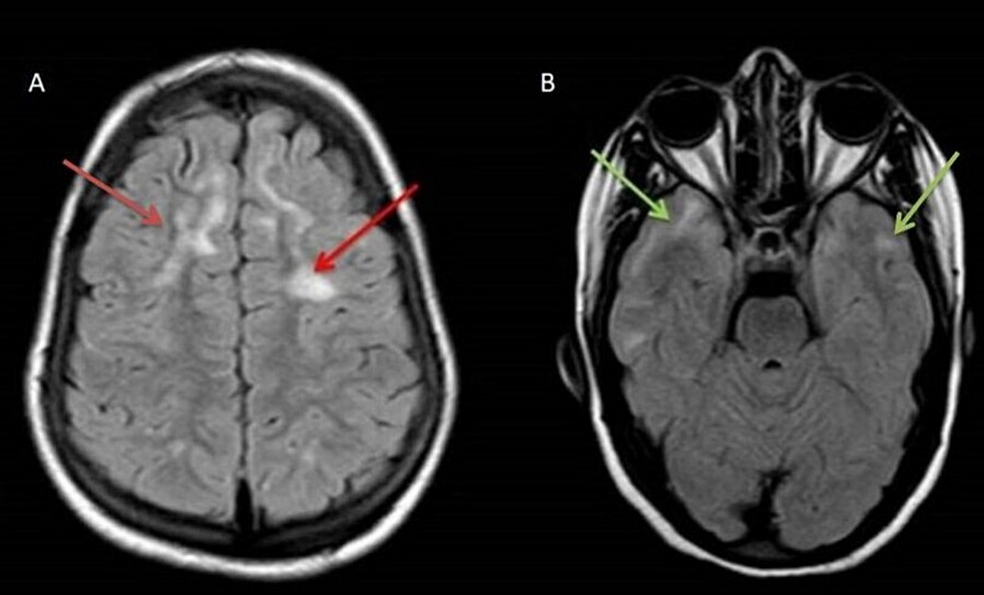
Axial fluid-attenuated inversion recovery (FLAIR) images (A) Confluent hyperintensities involving the white matter in B/L frontal lobes (red arrows). (B) Similar confluent hyperintensities involving white matter in B/L temporal lobes (green arrows). B/L: bilateral.

MRI brain showed discrete as well as confluent T2/FLAIR hyperintense lesions seen in the subcortical and deep white matter of bilateral frontal, temporal and parietal lobes (Figure 5A, 5B).

**Figure 6:**
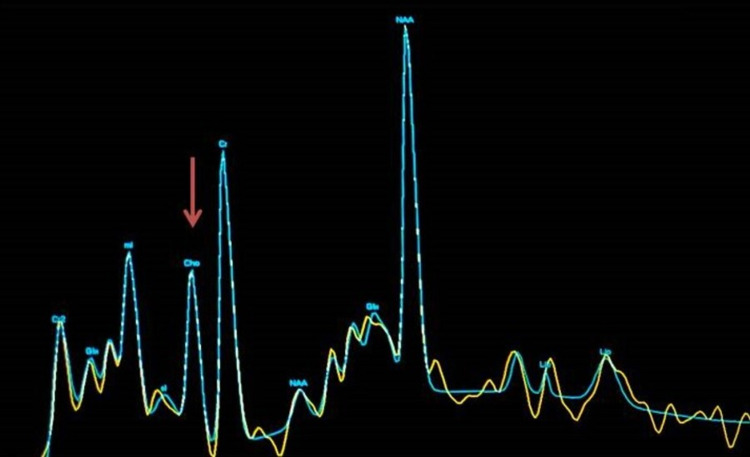
Proton magnetic resonance spectroscopy (TE: 144 ms) Mildly reduced choline (Cho) levels are seen in the affected areas (red arrow) with a choline/creatine (Cho/Cr) ratio of 0.5. NAA: N-acetylaspartate, Glx: glutamate-glutamine, Lip: lipid, mI: myo-inositol, TE: time to echo.

These lesions showed no diffusion restriction on diffusion-weighted imaging (DWI)/apparent diffusion coefficient (ADC) images and no post-contrast enhancement. No blooming was seen on gradient images. On magnetic resonance spectroscopy, mildly reduced choline and NAA levels were seen in these lesions (Figure 6).

## Discussion

SSPE was first described as an inclusion body encephalitis by Dawson in 1934. It is encountered worldwide and is relatively more frequently seen in the Middle East [4]. The disease onset is typically after a decade of asymptomatic period of measles infection. The susceptibility to this disease is more when measles develop during early years of life.

Symptom onset is usually from four to 23 years of age [1]. The disease becomes symptomatic at around nine years from the point of contracting the infection [1]. Initially, the presentation is usually subtle and includes behavioural changes and mild mental deterioration with no specific signs on neurological examination. As the disease progresses, non-specific symptoms like myoclonic jerks develop. It initially involves the head followed by the trunk and limbs. It can also present as gait difficulties, episodes of head drop and falling. Few patients may also manifest motor symptoms like ataxia and dystonia. Generalised tonic-clonic seizures and partial seizures have also been reported in some [3].

Neuroimaging rarely helps to diagnose SSPE in its early course. In the early course of the disease, computed tomography of brain is usually normal with diffuse cerebral oedema becoming evident only in its later stages [3]. White matter abnormalities are better appreciated on MRI. However, MRI findings in SSPE are not pathognomonic [1]. Previous studies on subacute sclerosing panencephalitis have shown typical bilaterally asymmetric hyperintensities in parietal and temporal lobes. Over time, more confluent lesions can be seen involving the periventricular white matter, corpus callosum and basal ganglia [5]. Corpus callosum is involved as an extension of the posterior deep white matter abnormality rather than its primary involvement. Later on cerebral atrophy sets in. Early SSPE has a predilection for the posterior areas of brain especially the parieto-occipital and posterotemporal regions [6]. One study found that the disease process starts in the occipital white matter and proceeds anteriorly [7]. In our study, frontal white matter was involved in both the cases. Involvement of the brain stem is known to be rare [8]. Grey matter is generally spared even in advanced stages of the disease in majority of cases. However, a study performed on 15 patients observed early involvement of the grey matter [2].

Though usually the lesions in SSPE demonstrate no mass effect or post-contrast enhancement, it has been reported by some authors in few patients especially early in the course of the disease [9]. No post-contrast enhancement of lesions was seen in our study.

Subacute sclerosing panencephalitis shows loss of neurons, gliosis and inflammation on histopathologic examinations [3]. Several proton magnetic resonance spectroscopy (MRS) studies on SSPE have reported marked decrease in NAA peaks. In addition, increase in choline, myo-inositol peaks and presence of lactate doublet wave were also noted. Decrease in NAA, increase in choline and the presence of lactate can be ascribed to neuronal loss, demyelination and macrophagic infiltration, respectively [10,11]. In our study, NAA was reduced in both the cases on MRS.

Although MRI may show changes in relation to disease duration, it does not correlate with the disease severity. However, MRI can help in pointing towards the diagnosis in the typical clinical settings and also by ruling out any other neurological entity like tumours and other infective/demyelinating entities while CSF reports are awaited.

At present, no cure exists for the disease. Treatment comprises supportive therapy for the management of seizures and other complications. Sodium valproate is a commonly used anti-epileptic agent [3]. Anti-viral drugs and immunomodulators have been used singly or in combination to slow down the progression of the disease. Currently, the most effective means of eradicating this disease is preventing measles infection through adequate and active immunisation.

## Conclusions

SSPE is usually diagnosed based upon typical clinical features, characteristic EEG changes and presence of raised anti-measles antibodies in the plasma and in cerebrospinal fluid of the patient. MRI brain is an adjunctive modality, revealing white matter hyperintense lesions which may be subacute or chronic, but helps in early diagnosis while the CSF reports are awaited and also in ruling out other brain pathologies like tumours or other neurodegenerative/acute or chronic aetiologies. However, MRI findings in SSPE are not pathognomonic, and MRI can be normal in patients of early SSPE.
